# Enhanced Mechanical Properties of PVA Hydrogel by Low-Temperature Segment Self-Assembly vs. Freeze–Thaw Cycles

**DOI:** 10.3390/polym15183782

**Published:** 2023-09-15

**Authors:** Fei Wu, Jianfeng Gao, Yang Xiang, Jianming Yang

**Affiliations:** 1 Taiyuan Institute of Technology, Taiyuan 030008, China; wufeikitty0311@163.com (F.W.); gao211@163.com (J.G.); 2 State Key Laboratory of Dynamic Measurement Technology, School of Instrument and Electronics, North University of China, Taiyuan 030051, China; 3 Shanxi Province Key Laboratory of Functional Nanocomposites, College of Materials Science and Engineering, North University of China, Taiyuan 030051, China; 4 Research Center for Engineering Technology of Polymeric Composites of Shanxi Province, North University of China, Taiyuan 030051, China; 5 School of Chemistry and Chemical Engineering, Anhui University of Technology, Ma’anshan 243032, China; 6 State Key Laboratory of Molecular Engineering of Polymers, Fudan University, Shanghai 200438, China

**Keywords:** PVA, hydrogel, mechanical properties, segment self-assembly, freeze–thaw

## Abstract

The rapid and effective fabrication of polyvinyl alcohol (PVA) hydrogels with good mechanical properties is of great significance yet remains a huge challenge. The preparation of PVA hydrogels via the conventional cyclic freeze–thaw method is intricate and time-intensive. In this study, a pioneering approach involving the utilization of low-temperature continuous freezing is introduced to produce a novel PVA-ethylene glycol (EG) gel. Fourier transform infrared (FTIR) spectroscopy, X-ray diffractometry (XRD) and scanning electron microscopy (SEM) confirm that with the assistance of EG, PVA molecular chains can self-assemble to generate an abundance of microcrystalline domains at low temperatures, thus improving the mechanical properties of PVA-EG gel. Remarkably, when the mass ratio of H_2_O/EG is 4:6, the gel’s maximum tensile strength can reach 2.5 MPa, which is much higher than that of PVA gels prepared via the freeze–thaw method. The preparation process of PVA-EG gel is simple, and its properties are excellent, which will promote the wide application of PVA tough gel in many fields.

## 1. Introduction

Polyvinyl alcohol (PVA) hydrogels have been widely developed due to their inherent non-toxicity and excellent biocompatibility in applications such as 3D printed materials [1], sensors [2], artificial biological soft tissue [3], flexible wearable electronics [4] and implantable biomedical devices [5]. At the molecular level, the creation of PVA hydrogels typically involves cross-linking PVA molecular chains to achieve water retention while maintaining insolubility in water [6,7]. To accomplish this, PVA molecular chains are commonly cross-linked using multi-functional chemical crosslinking agents, such as formaldehyde, glyoxal, glutaraldehyde, etc., but these crosslinking agents are basically toxic. Ceylan et al. have found that MEF cells cultured on a glutaraldehyde chemically cross-linked PVA scaffold exhibited a change in cell morphology from flat to round, accompanied by contraction and foaming on the cell surface [8]. These changes displaying early apoptosis features suggest that chemically crosslinked PVA had a cytotoxic effect on cells. Thus, when PVA gels are used in direct contact with biological tissue and fluids, we must consider the cytotoxicity of gel crosslinkers because toxic crosslinkers may penetrate into biological tissues via solvent exchange [9]. Therefore, chemical crosslinking has seriously hindered the application of PVA hydrogels in biomedicine [10,11].

Peppas first reported the preparation of physically crosslinked PVA gels via the cyclic freeze–thaw (FT) method (hereafter shortened as PVA-FT hydrogels), in which the microcrystalline region of PVA acts as the physical crosslinking point of the hydrogels [12]. The interchain and intra-chain hydrogen bond interactions of PVA molecules play a key role in the construction of microcrystalline regions [13]. The preparation process, including such variables as the concentration of PVA solution, freezing and thawing times and freeze–thaw times, will affect the size and number of microcrystalline zones and ultimately affect the macroscopic properties of the hydrogel [14]. However, the multi-freeze–thaw method is more complicated, time-consuming and laborious, and the hydrogel strength obtained at lower PVA concentration is usually low, resulting in difficulties in application [15]. In recent years, numerous innovative approaches have emerged to enhance the strength of physically crosslinked PVA gels, building upon the foundation of the FT method. For instance, anisotropic gels are obtained by employing an ice template, which induces the alignment of PVA chains parallel to the ice crystal direction [16]. Wu et al. employed the freezing–soaking technique, immersing frozen PVA solutions in salt solutions. Via the salting-out effect, they fine-tuned the aggregation level of PVA chains at the molecular scale to create ultra-tough PVA hydrogels [17]. Gao’s group introduced a novel wet-annealing strategy, which fosters a free and unrestricted environment for the mobility of PVA molecules, facilitating the construction of mechanically robust polymer networks [18]. Compared to the conventional dry-annealing method, the wet-annealing approach grants PVA chains greater latitude for conformational adjustments, resulting in the formation of a more densely interconnected hydrogen bond network [19]. However, it is worth noting that the preparation processes associated with these methods are often intricate and challenging. Therefore, it is imperative to seek simpler approaches for the fabrication of high-strength PVA gels.

Ethylene glycol (EG) is widely employed as an antifreeze agent in industrial water systems. Its freezing point is about −12 °C; when mixed with water, EG readily forms robust hydrogen bonds with water molecules, leading to a significant reduction in the saturated vapor pressure of water [20]. When the concentration of EG is adjusted, the freezing point of the binary mixture of EG and water can be reduced to about −70 °C. Yu’s group successfully synthesized high-strength, high-toughness PVA/EG gels by dissolving PVA in a binary mixture of EG/H_2_O [21]. However, the preparation process still relied on the cyclic freeze–thaw method, and the untapped potential of the EG/H_2_O mixture as a solvent for PVA remains to be comprehensively explored. Thus, a deeper investigation into the underlying mechanisms governing the formation, reinforcement and toughening of PVA/EG gels is imperative. This endeavor holds promise for advancing the creation of PVA gels endowed with superior mechanical properties. 

In this study, PVA-EG gels were prepared via the low-temperature chain segment self-assembly method, and their structure and properties were compared with those prepared via the common cyclic freeze–thaw method; the comparison confirmed that the gel prepared via low-temperature chain segment self-assembly had better mechanical properties. Finally, the mechanisms of EG-strengthened gel was discussed.

## 2. Materials and Methods

### 2.1. Materials

PVA (M_n_ = 7.7 × 10^4^, 98–99% hydrolysis degree) and EG (moisture ≤ 0.2%, A.R.) were purchased from Aladdin Biochemical Technology Co., Ltd., (Shanghai, China). All reagents were used directly without further purification.

### 2.2. Methods

#### 2.2.1. Preparation of PVA-EG and PVA-FT Gels

A certain amount of PVA was dissolved in a mixed solvent of deionized water and EG with different mass ratios. In order to study the strengthening advantage of EG on PVA gels at low concentrations, the concentration of all PVA gels in this study was 10 wt.%. The mixture was mechanically stirred for 2 h at 95 °C to completely dissolve the PVA. After that, the mechanical stirring was stopped and slowly cooled, so that the bubbles in the solution gradually rose and burst. The solution was then refrigerated (−18 °C) for a certain amount of time to obtain PVA-EG gels. The PVA-FT gels were prepared using the above procedure in the absence of EG. In addition, PVA-FT gels underwent freezing and thawing only once; if the PVA-FT gels underwent multiple freeze–thaw cycles, it was named PVA-FT-n, where n indicates the number of freeze–thaw cycles, and the thawing method was to leave the gels at room temperature for 5 h.

#### 2.2.2. Characterizations

For more effective comparative analysis, PVA-EG gels with mass ratio of H_2_O/EG of 6:4 were soaked in excessive deionized water; this was repeated 3 times to fully replace the EG inside the gels, and then the gels were freeze-dried to obtain the sample to be tested. The PVA-FT gels were freeze-dried directly for the test. The obtained freeze-dried sample was cut into 2 cm × 2 cm pieces for direct FTIR, DSC and XRD tests. 

The FTIR absorption spectra of the samples were measured via Fourier transform infrared spectroscopy (FT-IR, Nicolet iS50, Waltham, MA, USA) in attenuated total reflection (ATR) mode, and the peak fitting was performed using Peakfit software (Version 4.0). 

X-ray diffractometry (XRD, HAOYUAN DX-2700B, Dandong Haoyuan Instrument Co., Ltd., Dandong, China) was used to analyze the crystal structures of samples in the 2*θ* range of 5–80°, and the crystallinity was calculated with MDI Jade software (Version 6.5). The crystallite size (*D*) of the gels was calculated from the following equation:

(1) $$D=\frac{0.89\lambda }{\beta \mathrm{Cos}\theta }$$

where *λ* was the wavelength of the X-ray source, *β* was the full width at half maximum and *θ* was the diffraction angle. 

The DSC thermograph of the samples was measured via differential scanning calorimeter (DSC, TA DSC Q200, New Castle, DE, USA). In a typical DSC measurement, the gels were placed in a Tzero pan and heated up from −40 °C to 250 °C at a heating rate of 20 °C min^−1^ under a N_2_ atmosphere with a flow rate of 30 mL min^−1^. The crystallinity (*X*) in the dry gels would be calculated as:

(2) $$X=\frac{H_{crystalline}}{H_{crystalline}^{0}}$$

where $H_{crystalline}^{0}$ = 138.6 J g^−1^ was the enthalpy for fusing PVA with the 100 wt.% crystallinity measured at the equilibrium melting point; $H_{crystalline}$ was the integrating endothermic transition of the melting area of the crystalline domains of sample.

Freeze-dried samples were broken in liquid nitrogen to expose their internal morphology and observed via scanning electron microscope (SEM, Hitachi SU8010, Tokyo, Japan), and the as-prepared internal surface was coated with a layer of gold.

#### 2.2.3. Mechanical Tests

The samples were made into dumbbell shapes to test their mechanical properties. The dumbbell-shaped samples were prepared by pouring the PVA solution directly into the dumbbell-shaped molds and then freezing and thawing. The mechanical properties of gels were measured via universal testing machine (Instron Corporation, Waltham, MA, USA) equipped with a 100 N load cell at a crosshead speed of 50 mm/min, in accordance with the ISO standard (ISO 527-1: 1993). Five samples were tested for each experiment. The tensile stress (*σ_t_*) was calculated using the following equation:

(3) $$\sigma_{t}=\frac{Force}{Area}$$

where “*Force*” was the force applied to the sample by the universal testing machine; “*Area*” was the average cross-sectional area at the middle position of dumbbell shaped hydrogel.

The tensile strain (*ε_t_*) was calculated based on the following equation:

(4) $$\varepsilon_{t}=\frac{\Delta l}{l_{0}}$$

where Δ*l* was the relative elongation of samples; *l*_0_ was the gauge length of the specimen.

The tensile strength (*σ_b_*) and elongation (*ε_b_*) of samples were the tensile stress and tensile strain when the samples broke. The elastic modulus (*E*) was calculated within the initial linear range of the stress–strain curve. *E* was calculated using the following equation:

(5) $$E=\frac{\sigma_{t}}{\varepsilon_{t}}$$

The toughness of the samples was calculated from the areas under the tensile stress–strain curves.

## 3. Results and Discussion

### 3.1. Preparation Mechanism

**Figure 1:**
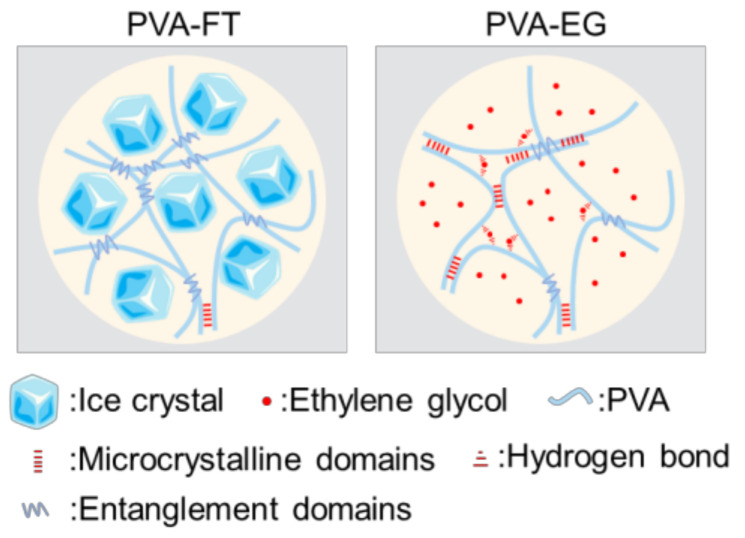
Microstructure diagram of PVA-EG and PVA-FT gel.

**Figure 2:**
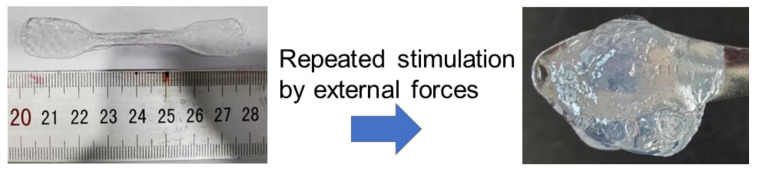
The morphology of PVA-FT gel under repeated stimulation via external forces.

The preparation mechanism of PVA gels obtained from two different processes is shown in Figure 1. For PVA-FT gel, the obtained PVA solution was frozen in the refrigerator. During this period, the water in the PVA solution separated out rapidly to form large solid-phase ice crystals [22]. Due to the reduction of the solvent, the molecular chains of PVA rapidly gathered and tangled together randomly, forming a large number of physical entanglement domains. PVA molecular chains could only self-assemble to form a small number of stable microcrystalline zones due to the fast excessive freezing rate. When the gel was thawed at room temperature, a large number of physical entanglements in the system were in an unstable state, and these entanglements were easily untangled via external stimulation. Experiments also confirmed that if 10 wt.% of PVA-FT gel underwent only one freeze–thaw cycle, it would return to a solution-like state under repeated external stimulation, as shown in Figure 2. Although the strength of PVA-FT gel could be gradually increased via repeated freeze–thaw cycles, as reported in the literature [23], this is generally time-consuming and laborious and not economical. For PVA-EG gel, after adding EG, the freezing point of the mixture of EG and water was significantly reduced. At the appropriate ratio, when the freezing point of the mixture was lower than the freezing temperature, the molecular chain segment of the PVA system could still move freely in the mixture, and the side group of the PVA molecular chains had a continuous and regular arrangement of hydroxyl groups. Self-assembly within or between molecular chains easily formed stable microcrystalline domains, which could still exist stably at room temperature. With the continuous extension of freezing time, the number of microcrystalline regions in the system increased. These microcrystalline regions acted as physical crosslinking points in the PVA gel system; in other words, the dense physical cross-linking network enables PVA gels to withstand greater external loads, which is consistent with the view of Zhao et al. that the number of microcrystalline regions controls the mechanical properties of gels [24]. In addition, some EG molecules also formed hydrogen bonds with PVA to connect adjacent PVA molecular chains and form cross-linking points, thus further enhancing the gel strength. However, as EG is a small molecule with strong mobility in aqueous solution, these cross-linking points were unstable and uncross-linked easily under external stimulation. In theory, the hydrogen bond between EG and PVA molecular chains is not the main factor of gel enhancement, which can also be verified from the following mechanical tests.

### 3.2. Structural Characterization

#### 3.2.1. FTIR and DSC Analysis

**Figure 3:**
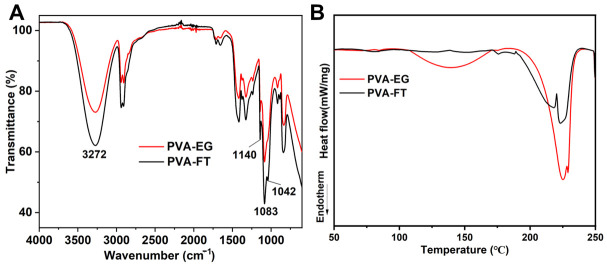
FTIR spectra (**A**) and DSC thermograph (**B**) of PVA-FT and PVA-EG gel.

The FTIR spectra of PVA-FT and PVA-EG gel are shown in Figure 3A. In Figure 3A, PVA gels showed the characteristic stretching bands of –OH at 3272 cm^−1^ [25]. The symmetric stretching vibration peak of O-C-C bond at 1140 cm^−1^ was related to the crystallization of PVA, and the antisymmetric stretching vibration peak of O-C-C bond at 1083 cm^−1^ and 1042 cm^−1^ belonged to the amorphous phase. Sugiura et al. used the absorbance ratio (D_1145_/D_1096_) to indicate the crystallinity of PVA [26]. In this study, when PVA-FT and PVA-EG were compared, it could be observed that the amorphous peak of PVA-EG at 1042 cm^−1^ was significantly weakened, indicating that the crystallinity of PVA-EG was greater than that of PVA-FT. To quantify crystallinity, DSC was employed to study the crystalline behavior of PVA-FT and PVA-EG gel (Figure 3B). The narrow peak present in the heat flow curve (190–240 °C) represented the melting range of the crystalline domains of dried gels. It was found that the crystallinity of PVA-FT and PVA-EG was 35.00% and 45.12%, respectively. This obviously indicated that with the aid of EG, PVA formed more microcrystalline domains, which significantly affected the strength of PVA-EG gel.

#### 3.2.2. XRD Analysis

**Figure 4:**
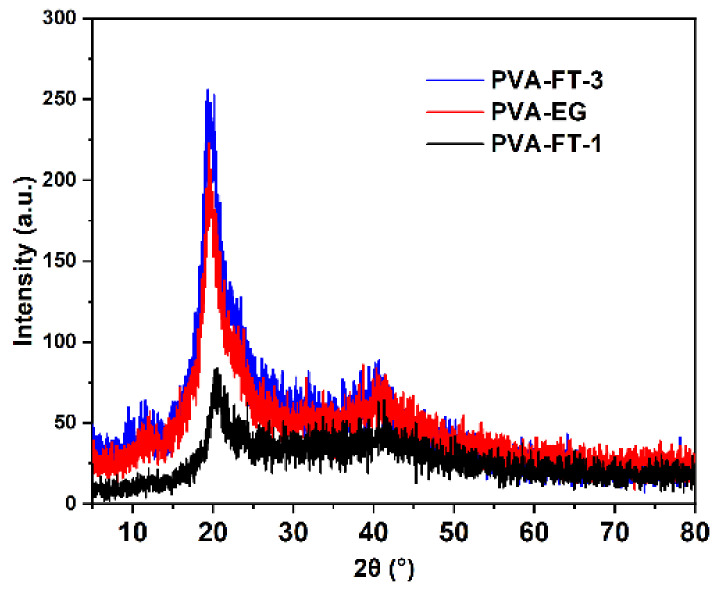
XRD pattern of PVA-FT and PVA-EG gel.

As shown in Figure 4, the diffraction peaks of all samples at 19.46° can be attributed to the semi-crystalline PVA hydrogel (101) crystal planes [27]. The diffraction peak strength of all samples was obviously different. The crystallinity of PVA-FT-1, PVA-FT-3 and PVA-EG was 26.01%, 57.86% and 48.38%, respectively, calculated via Jade software. The crystallinity of PVA-FT-1 was obviously lower than that of PVA-EG, which was consistent with the results obtained via FTIR. Only after three freeze–thaw cycles did the crystallinity of PVA-FT-3 become greater than that of PVA-EG. Furthermore, the average grain size of the gel samples was calculated using the Scherrer formula, as shown in Table 1. Interestingly, the average crystallite size of PVA-FT-3 was basically the same as that of PVA-FT-1, indicating that multiple freezes did not increase the average crystallite size of PVA microcrystalline domains but only increased the number of crystallites. However, the average crystallite size of the PVA-EG was significantly larger than that of the PVA-FT, indicating that with the help of EG, the PVA had a larger chain segment mobility in the supercooled state and could assemble into larger crystalline size with time. In addition, the larger crystalline size in PVA-EG should have had a significant effect on the mechanical properties, which was confirmed by later studies.

#### 3.2.3. SEM Analysis

**Figure 5:**
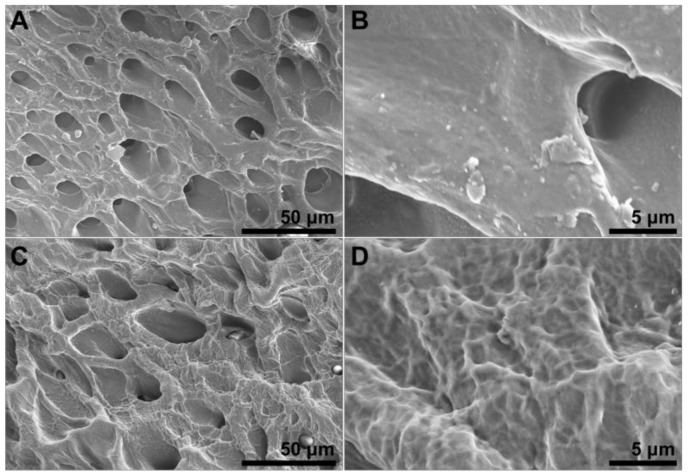
Microstructure of PVA-FT (**A**,**B**) and PVA-EG (**C**,**D**) gel.

As shown in Figure 5A, the PVA-FT gel was composed of continuous micropores with an average diameter of about 20 μm, and the pore walls of these micropores were smooth and wrinkle-free (Figure 5B). As shown in Figure 5C, the micropore size of PVA-EG gel was similar to that of PVA-FT, but there were many nanoscale folds in the pore wall (Figure 5D), indicating that the molecular chains of PVA gel will strongly aggregate to form nanoscale microcrystalline domains under the inducement of EG, thus ultimately forming a network of nanofibers on the surface of the micrometer-scale pore wall. The high aggregation and crystallization of PVA chains caused by the repulsion of PVA chains and low-concentration solvent (EG) further enhanced the pore wall [28]. The hierarchical structure had a profound effect on the mechanical properties of the PVA-EG gel [29].

### 3.3. Mechanical Properties

**Figure 6:**
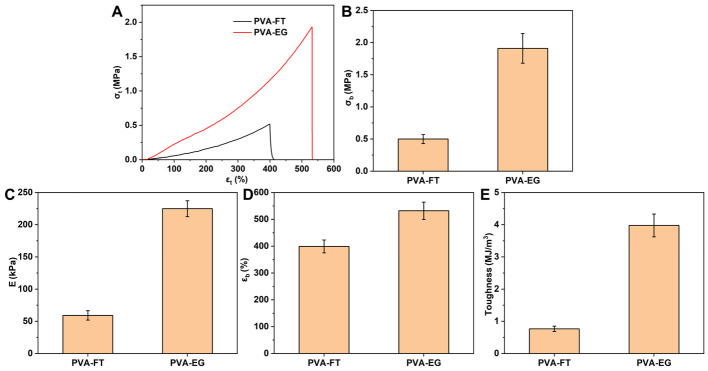
Typical *σ_t_*–*ε_t_* curves (**A**), *σ_b_* (**B**), *E* (**C**), *ε_b_* (**D**), and toughness (**E**) of PVA-FT gel and PVA-EG gel (mass ratios of H_2_O/EG of 6:4).

Compared with the PVA-FT gel, the PVA-EG gel had better mechanical properties. As shown in Figure 6, when the total freezing time was 30 h (wherein PVA-FT was frozen for 10 h and thawed for 5 h, and the cycle repeated 3 times, while the PVA-EG gel was only continuously frozen for 30 h), the PVA-FT gel exhibited lower *σ_b_*, *E*, *ε_b_*, and toughness, which were 0.50 MPa, 59.1 kPa, 399%, and 0.77 MJ/m^3^, respectively. The *σ_b_*, *E*, *ε_b_*, and toughness of PVA-EG gel were 1.91 MPa, 224.9 kPa, 532%, and 3.98 MJ/m^3^, respectively; its *σ_b_*, *E* and toughness were 3.8, 3.8 and 5.2 times of that of PVA-FT gel, respectively. This showed that the addition of EG greatly improved the mechanical properties of the PVA gel.

**Figure 7:**
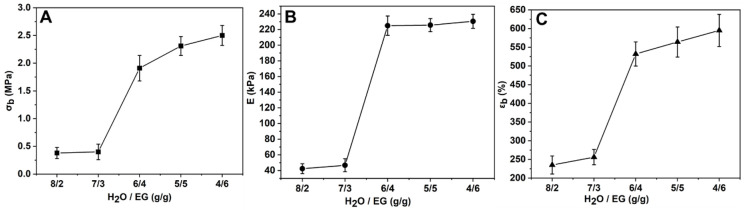
The *σ_b_* (**A**), *E* (**B**) and *ε_b_* (**C**) of PVA-EG gels with different H_2_O/EG mass ratios (freezing continuously for 30 h).

The mass ratio of water and EG affected the freezing point of the binary mixture, and then the self-assembly efficiency of PVA molecular chain [30]. Therefore, the effect of water and EG mass ratio on the mechanical properties of PVA-EG gel was further studied. As EG is a poor solvent for PVA, when the mass ratio of H_2_O/EG is lower than 3:7, PVA is difficult to completely dissolve into a homogeneous solution, and a large number of bubbles in the system are extremely difficult to remove. Therefore, the effects of mass ratios of H_2_O/EG of 8:2, 7:3, 6:4, 5:5 and 4:6 on the mechanical properties of PVA-EG gel were studied. When the mass ratio of H_2_O/EG was 8:2, 7:3, 6:4, 5:5 or 4:6, the freezing temperature of the mixture was −7.8 °C, −14 °C, −22.3 °C, −33.7 °C or −40 °C, respectively. Since the freezing temperature of the refrigerator is −18 °C, when the mass ratio of H_2_O/EG was 8:2 or 7:3, the mixture of EG and water in PVA-EG gel froze in a short time, and the experiment also confirmed that the PVA-EG gel completely froze within 4 h. It can be seen from Figure 7 that when the mass ratio of H_2_O/EG was 8:2 or 7:3, the resulting gels’ *σ_b_*, *E* and *ε_b_* were 0.38, 0.4 MPa, 42.3, 46.7 kPa, 235, 256%, respectively, which is significantly lower than that of PVA-EG gels with other mass ratios. This showed that when the freezing temperature was below the freezing point of the EG and water mixture, the rapid freezing rate of the mixture left too little time for the PVA molecular chain to self-assemble to form a sufficient number of microcrystalline domains structures and significantly reduced the mechanical properties of the gel.

**Figure 8:**
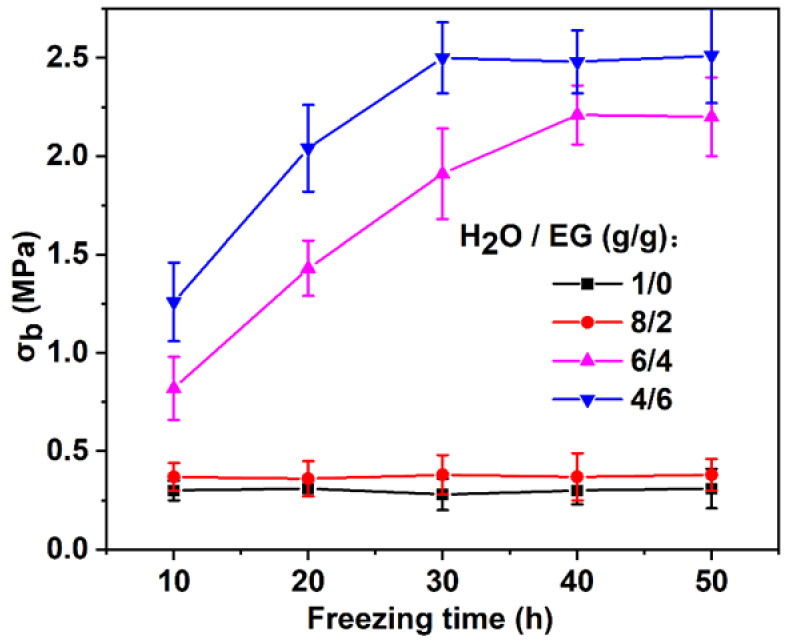
The *σ_b_* of PVA-FT and PVA-EG gels with different H_2_O/EG mass ratios.

Interestingly, when the mass ratio of H_2_O/EG was 8:2 or 7:3, the mechanical properties of the gel were also significantly lower than those of the PVA-FT gel (Figure 6), which may be due to the fact that the mechanical strength of the gel did not significantly improve after the freezing time was increased after the complete freezing of PVA gel. Therefore, in order to verify this hypothesis, the effects of different freezing time on PVA-EG gels were studied. As can be seen from Figure 8, when the mass ratio of H_2_O/EG is 1:0 or 8:2, *σ_b_* remained at 0.30 and 0.36 MPa, respectively, during the freezing time from 10 to 50 h, and the gel strength of the PVA-EG (mass ratios of H_2_O/EG of 8:2) was slightly greater than that of the PVA-FT (mass ratios of H_2_O/EG of 1:0), but the difference was not obvious. When the mass ratio of H_2_O/EG was 6:4, with the extension of freezing time, the *σ_b_* increased gradually, and the *σ_b_* remained at about 2.2 MPa after 40 h. Interestingly, when the mass ratio of H_2_O/EG was 4:6, *σ_b_* reached a maximum value of about 2.5 MPa when the freezing time reached 30 h. This indicated that the larger the mass ratio of H_2_O/EG, that is, the smaller the amount of EG added, the longer the freezing time PVA-EG gel required to reach maximum gel strength. This may be because the higher the freezing point of the mixture of EG and water, the worse the molecular chain mobility of PVA, making it less conducive to the formation of stable microcrystalline domains through self-assembly. In addition, it was found that the larger the amount of EG added, the higher the maximum gel strength of the PVA, but the difference was not significant. This may be due to the fact that the strong hydrogen bonding between EG and PVA chains created additional physical cross-linking points to further improve the mechanical properties of the gel [31].

**Figure 9:**
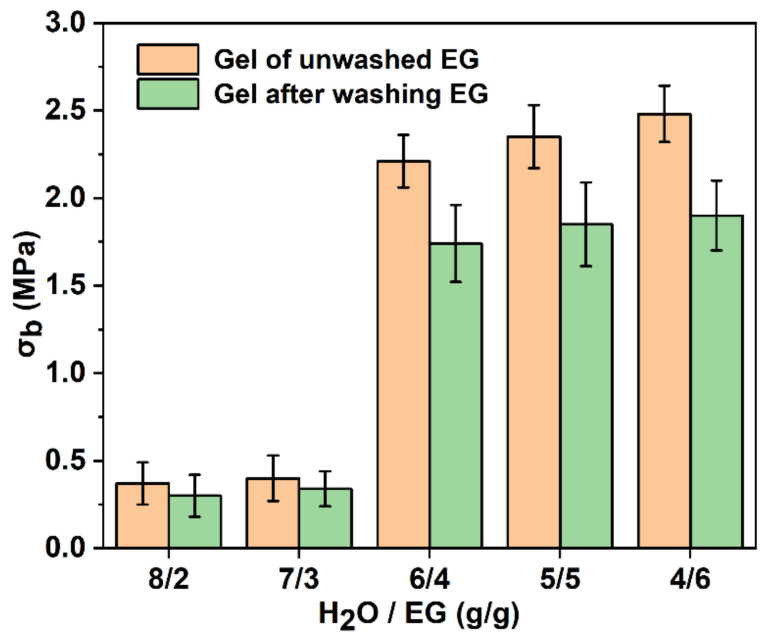
The σ_b_ of PVA-EG gels (freezing continuously for 40 h) before and after EG removal.

To further verify the main factors affecting the mechanical properties of the gel, the PVA-EG gel was fully placed in excess deionized water, the EG inside the gel was fully removed, and the gel strength before and after EG removal was tested, as shown in Figure 9. After cleaning, the σ_b_ from different H_2_O/EG mass ratios decreased compared with that from before cleaning, but it was still significantly higher at 6/4, 5/5 and 4/6 than that at 8/2 and 7/3 overall, which obviously indicated that the key factor affecting the gel strength of PVA-EG was the quantity of microcrystal region formed via PVA self-assembly rather than the hydrogen-bonding between EG and PVA. Therefore, in some application scenarios where EG is not required, this method could be used to remove EG in PVA-EG gel without much loss of mechanical properties. In addition, the ratios of 8/2 and 7/3 before and after cleaning were not significantly different, and the ratios of 6/4, 5/5 and 4/6 were significantly reduced. This indicated that when the amount of EG was small, EG did not significantly change the structure of the PVA gel. When the amount of EG was increased, in addition to inducing the self-assembly of PVA chains to form more stable microcrystal regions, the hydrogen-bonding between EG and PVA molecular chain also significantly improved the strength of the PVA-EG gel.

### 3.4. EG-Induced Strengthening Mechanism

**Figure 10:**
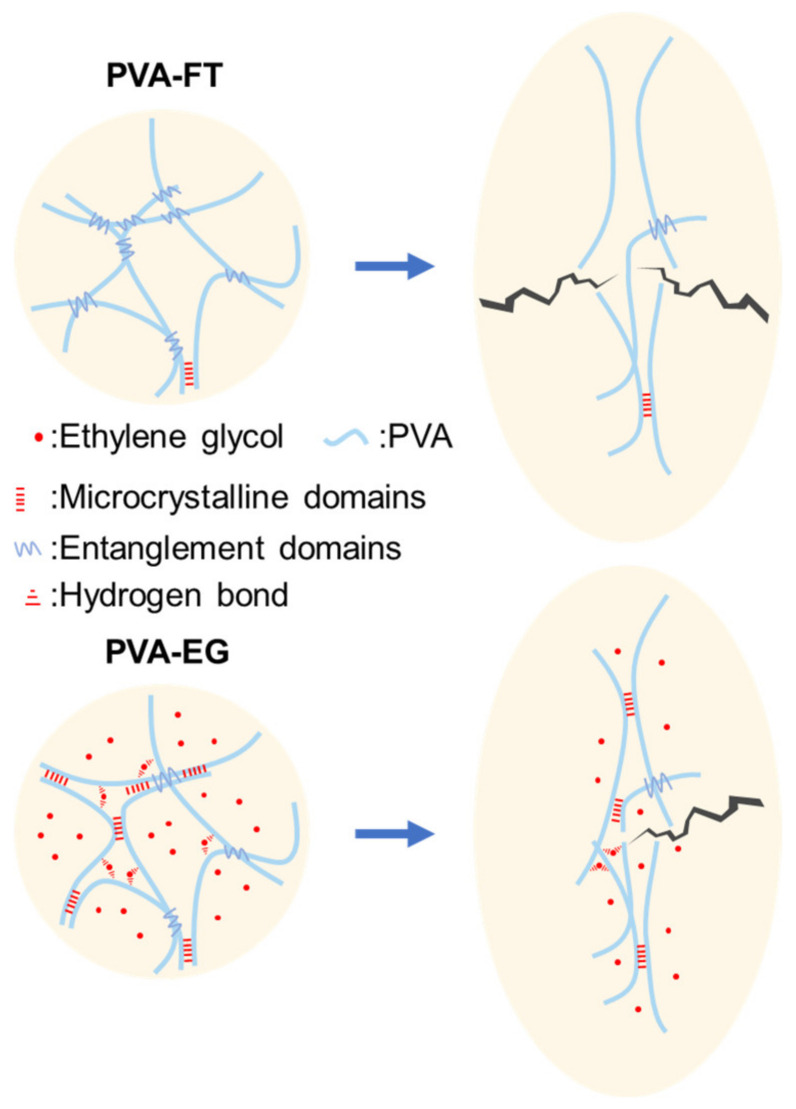
EG induced strengthening mechanism.

Figure 10 further illustrates the strengthening mechanism of PVA-EG gel. The interior of PVA-FT gel primarily comprised physical entanglement among PVA molecular chains. When tension was applied, the molecular chain segments gradually straightened out, the physical entanglement was untangled, and a large number of PVA molecular chains were free to form islands, resulting in low PVA chain concentration in some areas of the gel, making them susceptible to cracking even under relatively low tension, eventually leading to gel fracture and damage. As for the PVA-EG gel, its interior was mainly microcrystalline regions formed by via self-assembly of hydrogen bonds between PVA molecular chains. Under the action of external forces, the PVA molecular chain segments gradually straightened, and these microcrystalline regions would not be destroyed first, but would act as stable physical crosslinking points to improve the overall strength and stiffness. In addition, the hydrogen bond force formed from a small amount of EG and PVA molecular chain further improved the tensile properties of the gel. When the tension was further increased, the PVA molecular chain was pulled apart and the gel was eventually destroyed. Therefore, by reducing the freezing point of the gel system via EG, PVA molecular chains can be hydrogen bonded to self-assemble into stable microcrystalline regions at low temperatures, which can significantly improve the mechanical properties of PVA gels. Follow-up studies can also explore the effects of other antifreeze liquids such as methanol, ethanol and glycerol on the mechanical properties of PVA gels.

## 4. Conclusions

In summary, we have developed a very simple, versatile and novel method of preparing tough PVA hydrogels. By incorporating EG, we effectively lowered the freezing point of the mixed solvent, enabling PVA molecular chains to autonomously assemble into an abundance of microcrystalline domains at low temperatures, thereby improving the mechanical properties of PVA gel (tensile strength, elongation at break and modulus). Significantly, when the mass ratio of H_2_O/EG was lower than 6:4, the mechanical properties of PVA-EG gel increased rapidly. Especially when the mass ratio of H_2_O/EG was 4:6, *σ_b_* could reach a maximum of 2.5 MPa, a significant improvement over conventionally prepared PVA gels using freeze–thaw cycles. We foresee that with the help of EG, the originally weak PVA hydrogels can be applied in fields requiring materials with high mechanical properties, such as synthetic biological tissues, robotics and additive manufacturing. Considering that many hydrogel systems can form tough physical crosslinking domains via regular hydrogen bonding assembly, we are convinced that the presented strategy is not restricted to the systems presented here, but should also be applicable to other hydrogel systems, such as proteins and gelatin.

## Figures and Tables

**Table 1:** Diffraction peak and crystallite size of gels.

Samples	Diffraction Peak (2*θ*)	Crystallite Size (nm)
PVA-FT-1	20.62	2.67
PVA-FT-3	19.92	2.64
PVA-EG	19.94	2.76

## Data Availability

Not applicable.
